# Genetic characterization of novel fowl aviadenovirus 4 isolates from outbreaks of hepatitis-hydropericardium syndrome in broiler chickens in China

**DOI:** 10.1038/emi.2016.115

**Published:** 2016-11-23

**Authors:** Yanke Liu, Wenyan Wan, Dongsheng Gao, Yongtao Li, Xia Yang, Hongying Liu, Huixia Yao, Lu Chen, Chuanqing Wang, Jun Zhao

**Affiliations:** 1 Department of Preventive Veterinary Medicine, College of Animal Science and Veterinary Medicine, Henan Agricultural University, Zhengzhou 450002, Henan Province, China

**Keywords:** genetic properties, novel fowl aviadenovirus 4, hepatitis-hydropericardium syndrome, outbreak, amino-acid mutation

## Abstract

Since May 2015, severe outbreaks of hepatitis-hydropericardium syndrome (HHS) associated with infections of fowl aviadenovirus (FAdV) have emerged in broiler chickens in several Chinese provinces. To identify the genotype and gain a better understanding of the genetic properties of the FAdV strains responsible for the recent HHS outbreaks in China, the complete genome sequences of five isolates from outbreaks of HHS in broiler chickens in five provinces were determined. The results demonstrated that a novel fowl aviadenovirus 4 (FAdV-4) genotype was epidemic in China. To investigate the molecular characteristics of these Chinese FAdV-4 isolates, their genome contents were compared with those of reported pathogenic and non-pathogenic FAdV-4 strains. The comparative analysis revealed that the novel Chinese FAdV-4 isolates contain various genomic deletions and multiple distinct amino-acid mutations in their major structural genes. Two additional putative genetic virulence markers in the fiber 2 gene were identified. These findings confirmed some of the genetic differences between the pathogenic and non-pathogenic FAdV-4 isolates. The data presented in this report will enhance the current understanding of the molecular epidemiology and genetic diversity of FAdV-4 isolates in China and will provide additional insight into the critical factors that determine the pathogenicity of FAdV-4 strains. Finally, the emergence of this novel and highly pathogenic FAdV-4 genotype emphasizes that preventive measures against FAdV-4 infections on poultry farms should be implemented in China.

## INTRODUCTION

Adenoviruses are common infectious agents in poultry and other animals worldwide. The adenovirus family has been separated into five genera: *Mastadenovirus*, *Aviadenovirus*, *Siadenovirus*, *Atadenovirus* and *Ichtadenovirus* by the International Committee on Taxonomy of Viruses.^1^ Within the genus *aviadenovirus*, the important fowl aviadenoviruses (FAdVs) are separated into five species, designated A–E, based largely on molecular criteria, particularly the restriction enzyme fragmentation patterns and sequencing data.^1^ Viruses within each species can also be subdivided further into serotypes based on the results of serum cross-neutralization tests.^2^ FAdVs have been associated with a number of clinical diseases such as inclusion body hepatitis (IBH), hepatitis-hydropericardium syndrome (HHS), gizzard erosion, and other symptoms in chickens and other birds.^3, 4, 5, 6^ Of the various disease conditions associated with FAdVs in poultry, IBH and HHS are the most important. Many different FAdV serotypes have been associated with naturally occurring outbreaks of IBH. The previously reported viruses inducing IBH in geographically distinct regions belong mainly to the species *Aviadenovirus D* or *Aviadenovirus E*, in which serotypes 2, 3, 6, 7, 8a, 8b, 9, and 11 are combined.^7, 8, 9, 10, 11, 12^

HHS was first reported in Pakistan in 1987, and subsequent outbreaks have also been recorded in India, Kuwait, Iraq, Japan, Korea, Russia, Mexico, and South and Central America, causing significant losses to the poultry industry.^3, 13, 14, 15, 16, 17, 18^ HHS affects mainly 3- to 6-week-old broiler chickens. The disease is characterized by 20 and 80% mortality, which starts at three weeks, peaks for four to eight days in weeks four and five, and then declines.^3, 19, 20^ HHS also rarely occurs in breeding and laying flocks, with lower mortality rates. The predominant gross lesion in cases of HHS is a hydropericardium, characterized by the accumulation of clear, straw-colored fluid in the pericardial sac. Other lesions include an enlarged and discolored liver with foci of hemorrhage and/or necrosis.^14, 17, 21, 22, 23^ Epidemiological studies of FAdVs isolated from cases of HHS in different countries have shown that the HHS can be mainly attributed to an infection with a FAdV-C virus, FAdV-4,^14, 16, 24, 25, 26^ even though other agents may also be involved.^27, 28, 29^

Until 2015, the prevalence of IBH and/or HHS in China was relatively low with only sporadic outbreaks; however, in May 2015, a remarkable increase in HHS outbreaks began in various broiler-producing provinces of China. Therefore, to identify and gain a better understanding of the epidemiology of the FAdV strains responsible for the recent HHS outbreaks in China, the complete genome sequences of five isolates from broiler chickens involved in HHS outbreaks in five provinces were determined, and their genomic contents were compared with other pathogenic and non-pathogenic strains from different geographical regions. The data provide new insights into the primary pathogens of HHS, as well as the factors determining FAdV strain pathogenicity.

## MATERIALS AND METHODS

### Clinical signs, post-mortem findings and histological examinations

The main clinical signs and post-mortem lesions presented by the affected broilers were recorded. For a histological examination, samples of livers and hearts from affected birds were fixed in formalin, embedded in paraffin wax and cut into sections. The sections were stained with hematoxylin and eosin and examined for lesions by light microscopy.

### Sample collection and virus isolation

Between May and November 2015, 196 liver samples were collected from 32 commercial broiler farms experiencing HHS in five Chinese provinces (Henan, Anhui, Shandong, Shanxi and Jiangsu). The detection and differentiation of FAdVs in the samples were achieved via PCR with primers for the conserved FAdV hexon gene region in all five species of FAdV (Forward 5′-CAA RTT CAG RCA GAC GGT-3′ and Reverse 5′-TAG TGA TGM CGS GAC ATC AT-3′) using a previously described method.^30^ The PCR products were sequenced by Sanger sequencing. Five FAdV-4-positive liver samples from birds at five broiler farms experiencing high morbidity and mortality in the five Chinese provinces were selected for virus isolation. Virus isolation and plaque purification were conducted using primary chicken embryo liver (CEL) cells from 11-day-old specific pathogen-free (SPF) chicken embryos. The presence of FAdV-4 was confirmed by PCR and sequencing.

### Sequencing

Viral DNAs were extracted from the lysates of CEL cells infected with one of the five FAdV-4 isolates using a QIAamp DNA Blood Mini Kit (Qiagen, Valencia, CA, USA) according to the manufacturer's instructions. Specific primers for genome sequencing, designed on the basis of the sequence of the FAdV-4-ON1 strain,^31^ were used to amplify overlapping DNA fragments spanning the entire viral genome (Supplementary Table S1).The amplicons were sequenced in both directions using an ABI 3730xl DNA Analyzer (Applied Biosystems, Carlsbad, CA, USA) by Sangon Biotech Co., Shanghai, China. The complete genome sequences of the five FAdV-4 isolates, which were assembled using the Lasergene sequence analysis software package (DNASTAR Inc., Madison, WI, USA), were deposited in GenBank.

### Sequence analysis

The nucleotide and deduced amino-acid sequences of the five FAdV-4 isolates and certain reference strains (Supplementary Table S2) were edited, aligned and analyzed with the MegAlign program, a part of the Lasergene software package. Phylogenetic trees for the complete genomes and for each major structural gene segment were constructed using the maximum-likelihood method with the general time-reversible nucleotide substitution model and bootstrap test of 1000 replicates in the MEGA 6.06 software (www.megasoftware.net).

## RESULTS

### Clinical signs, post-mortem findings and histological examinations

**Figure 1:**
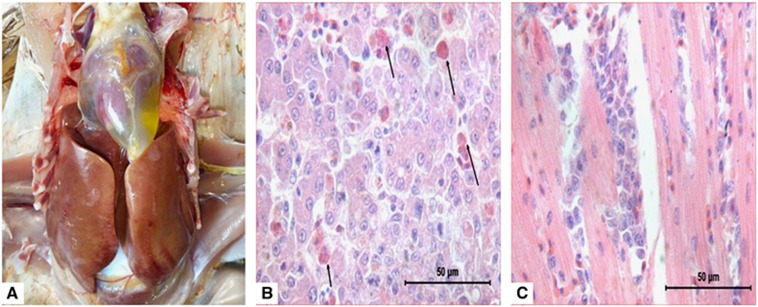
Postmortem and histological examinations of the liver and heart from affected birds with hepatitis-hydropericardium syndrome (HHS). (**A**) The accumulation of clear, straw-colored fluid in the pericardial sac and a discolored swollen liver with pinpoint hemorrhage. (**B**) Small multifocal areas of necrosis and basophilic intranuclear inclusion bodies in hepatocytes (H&E stain, original magnification × 400, scale bar=50 μm). (**C**) Lymphocytic infiltrates in association with myocarditis (H&E stain, original magnification × 400, scale bar=50 μm).

The HHS outbreaks were characterized by sudden occurrence, with a high mortality of up to 60% in 3- to 5-week-old broilers. The main clinical signs presented by the affected broilers included lethargy, diarrhea and prostration. Post-mortem examinations of the affected broilers exhibited typical HHS gross lesions, which were characterized by a flabby heart with an accumulation of straw-colored fluid in the pericardial sac and a discolored liver with necrotic foci. Upon histological examination, there were small multifocal areas of necrosis and mononuclear cell infiltration, including basophilic intranuclear inclusion bodies in hepatocytes and lymphocytic infiltrates in association with myocarditis (Figure 1).

### FAdVs in clinical samples and virus isolation

Using specific primers for the conserved FAdV hexon gene region in all five species of FAdV, a band of ~900 bp was amplified from all tested liver samples, indicating that the field samples were positive for FAdVs. The sequencing results of the PCR products indicated that the FAdV isolates in all samples were FAdV-4. The five FAdV-4-positive liver samples from affected birds caused similar CPE in primary CEL cells, characterized by the refractivity and ballooning of the cells and the detachment of the cultures from the flask in the form of bunches of grapes (data not shown).

### Genome sequencing and sequence analysis

**Figure 2:**
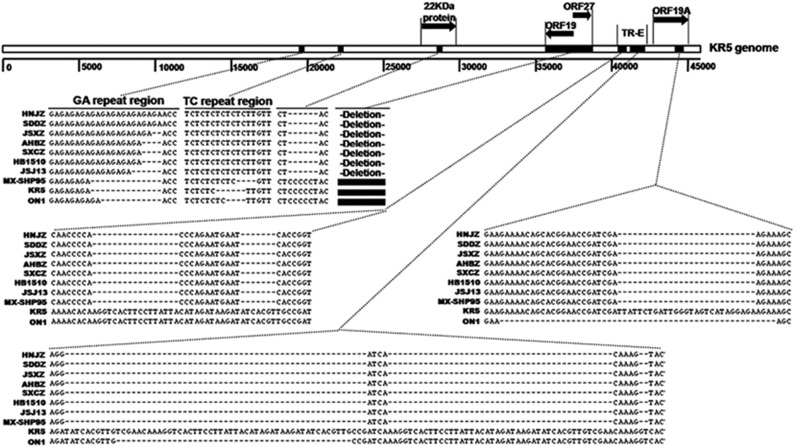
A comparison of the complete genomes among Chinese fowl aviadenovirus 4 (FAdV-4) strains and the previously reported sequences of highly pathogenic and non-pathogenic FAdV-4 strains. Insertions in the GA and TC repeat regions and deletions in the 22 kDa protein, ORF19, ORF27, ORF19A and tandem repeat (TR) E regions are shown. Open reading frames (ORFs).

The complete genomic sequences of isolate HNJZ from Henan, SDDZ from Shandong, SXCZ from Shanxi, AHBZ from Anhui, and JSXZ from Jiangsu were deposited in GenBank with accession numbers KU558760, KU558761, KU558762, KU569295 and KU569296, respectively. The full genome size of HNJZ, SDDZ, SXCZ, AHBZ and JSXZ was found to be 43 721, 43 721, 43 723, 43 725 and 43 725 bp in length, respectively. The G+C content (54.87% for HNJZ, SXCZ, JSXZ and AHBZ and 54.86% for SDDZ) was similar to those of other reported FAdV-4 strains, including ON1, KR5, JSJ13 and MX-SHP95 (54.63% to 54.88%). The gene order and shared open reading frames (ORFs) were similar to those of other reported FAdV-4 strains,^31, 32, 33, 34, 35^ except that there were deletions in the 22 kDa protein, ORF19, ORF27 and ORF19A (Figure 2).

The genomes of all five of the Chinese FAdV-4 isolates contain 42 predicted ORFs (Table 1). In common with FAdV-4 strains ON1 and KR5, homologs to the first ten ORFs (ORF0-ORF12) were also present in the left region of the genome of all five Chinese isolates.^31, 33^ In the central part of the genomes from the Chinese isolates, the gene organization was similar to that of other reported FAdV-4 strains. Two adjacent fiber genes, fiber 1 and fiber 2 were also present in the genomes of the Chinese isolates. Of the three hypothetical genes described for ON1, only the hypothetical 11.7 kDa gene was identified in the genomes of the Chinese isolates.^31^

It is notable that all the Chinese FAdV-4 strains have a 1966 bp deletion on the right end region of the genome compared with MX-SHP95, KR5 and ON1. The deletion is located at 35 413 bp to 37 378 bp compared to the nucleotide positions within the KR5 genome.^33^ It should be noted that during the preparation of this manuscript, this finding was published by another Chinese research group.^36^ However, we found that this deletion resulted in the absence of ORF19, ORF27 and ORF48. ORF20B observed in ON1 was merged with ORF20 to form a single ORF in all Chinese isolates and strains MX-SHP95 and KR5 due to a one base pair insertion at 34 769 bp in ON1. In addition, using the same inclusion criteria for ORF predication in ON1 and KR5, we also detected ORFs 42 and 49. However, one base pair deletion at 35 289 bp in ON1 resulted in a frameshift of ORF42 in all Chinese FAdV-4 isolates.

When compared with the non-pathogenic strains KR5 and ON1, all of the Chinese FAdV-4 strains and MX-SHP95 had the same deletion at the tandem repeat region (TR) E (41 804 to 41 862 bp and 41 867 to 41 910 bp) and ORF19A (44 492 to 44 518 bp) compared to the corresponding nucleotide positions within the KR5 genome (Figure 2). In the GA repeat region between gene pX and pVI, all of the Chinese isolates had longer GA repeats than the MX-SHP95, KR5 and ON1 strains. In the TC repeat region between the protease and the DNA-binding protein, all the Chinese isolates and MX-SHP95 had longer TC repeat regions than the non-pathogenic KR5 and ON1 strains. In ORF19A, which is close to the right end, all of the Chinese isolates have 830 amino acids, which is one amino-acid residue more than MX-SHP95, eight amino-acid residues more than ON1 (an eight amino-acid insertion in the Chinese isolates at position 723), and nine amino-acid residues less than KR5 (a deletion of nine amino-acid residues in the Chinese isolates at position 731).

The five Chinese isolates shared high genomic and major structural gene nucleotide identities with the recently reported Chinese FAdV-4, 100% identity with HB1510 (GenBank accession NO KU587519) and 99.9% identity with strain JSJ13.^32^ When compared with the FAdV-4 strains reported earlier, that is, the highly pathogenic MX-SHP95 strain, the non-pathogenic KR5 strain and the ON1 strain,^31, 33, 35^ nucleotide identities of 98.8%, 98.3% and 98.5% were observed for the complete genomes, 95.8%, 96.7% and 95.7% identities were observed for fiber 1, 97.2%, 95.9% and 95.8% identities for fiber 2, 98.6%, 98.9% and 98.7% identities for hexon, and 99.2%, 99.0% and 98.9% identities for the penton base.

Comparisons of the amino-acid sequences of the penton base, hexon, fiber 1 and fiber 2 with the corresponding structural proteins from the pathogenic strain MX-SHP95 and the non-pathogenic KR5 and ON1 strains revealed the presence of various amino-acid mutations (Table 2). Notably, all of the Chinese isolates and the virulent MX-SHP95 strain have the same amino-acid substitutions at position 42 (S to P) in the penton base protein, at 188 (I to R) in the hexon protein, at 432 (S to G) in the fiber 1 protein and at 219 (G to D), 300 (I to T), 305 (S to A), 307 (P to A), 378 (I to T), 380 (A to T), 435 (T to S) and 453(S to A) in the fiber 2 protein. The size of the fiber 1 protein in the Chinese FAdV-4 isolates is 431 amino acids, one amino acid less than MX-SHP95 and two amino acids less than KR5. Fiber 2 is 479 amino acids long in all of the Chinese isolates, the same as in strain KR5, but five amino acids more than the MX-SHP95 and ON1.

**Figure 3:**
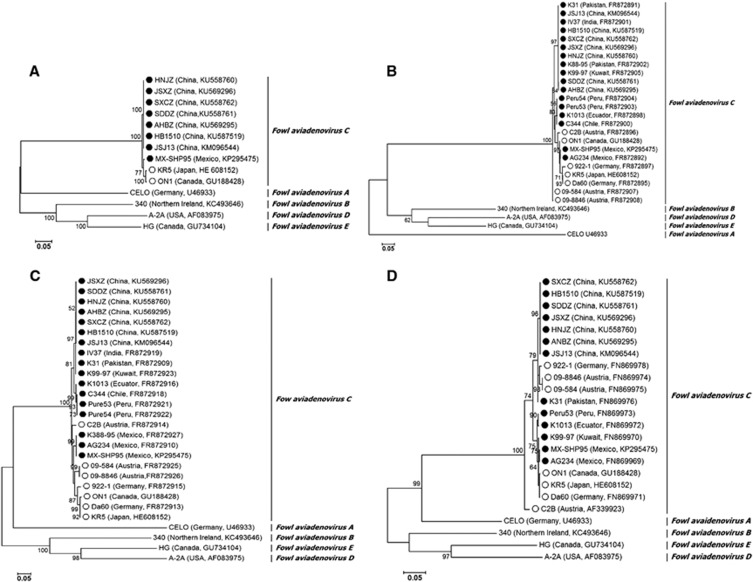
The results of phylogenetic analyses based on the nucleotide sequences of the currently available complete genomes (**A**), fiber 1 (**B**), fiber 2 (**C**) and hexon (**D**) gene sequences of FAdV-4 isolates and representative genes of other FAdV species. The trees were generated by the maximum-likelihood method with bootstrap tests of 1000 replicates using the MEGA 6.06 software. Bootstrap values are presented at key nodes. The scale bars indicate the nucleotide substitutions per site. Filled circles indicate HHS-inducing FAdV-4 strains. Open circles indicate non-HHS-inducing strains. Hepatitis-hydropericardium syndrome (HHS).

A phylogenetic analysis based on the complete genome showed that all Chinese FAdV-4 isolates clustered within FAdV-C together with other FAdV-4 isolates. Notably, the FAdV-C cluster was divided into two major groups. Group 1 consisted of two previously reported non-pathogenic strains, KR5 and ON1. Group 2 comprised all Chinese HHS FAdV-4 strains and the highly pathogenic MX-SHP95 strain (Figure 3A). FAdV-A and FAdV-C members have two fiber genes, fiber 1 and fiber 2,^31, 33, 37^ and our phylogenetic analysis based on the available complete fiber 1 and fiber 2 gene sequences also showed that all FAdV-C isolates clustered together. HHS-inducing FAdV-4 strains from Asia, South America and Mexico grouped together, whereas non-HHS-inducing strains from different geographical regions showed a closer evolutionary relationship (Figures 3B and 3C). A phylogenetic analysis based on the available hexon gene sequences did not show a strict clustering of the isolates according to involvement in disease. For example, non-HHS-inducing strains 922-1, 09-8846 and 09-584 grouped together with all Chinese HHS-inducing strains (Figure 3D). Remarkably, the Chinese isolates, together with 922-1, 09-8846 and 09-584, all had the same mutations at positions 164 (T to S), 238 (N to D), 240 (A to T), 243 (E to N), 263 (M to I) and 264 (I to V) of the hexon gene. A phylogenetic analysis based on the available complete penton base gene sequences showed the same cluster pattern as the genome data (data not shown).In addition, the closer genetic relationship between the FAdV-D and FAdV-E types was confirmed.^38, 39^

## DISCUSSION

Fowl aviadenoviruses cause different diseases of special importance in broiler chickens. Owing to the limited available information on the complete genome sequences of FAdVs, the factors determining their pathogenicity profiles remain unclear. Until now, the complete nucleotide sequences of FAdV genomes were only available for FAdV-1 (CELO virus, species FAdV-A), FAdV-4 (strains ON1, KR5, JSJ13, MX-SHP95, HB1510), FAdV-5 (strain 340, FAdV-B), FAdV-8 (strain HG, FAdV-E) and FAdV-9 (strain A-2A, FAdV-D).^31, 32, 33, 37, 38, 39, 40^ In the present study, the whole-genome sequences of five highly pathogenic FAdV-4 isolates have augmented the current knowledge of FAdV genomes and their pathogenesis.

The genomes of the Chinese FAdV-4 isolates differed in size from the previously reported highly pathogenic and non-pathogenic FAdV-4 strains.^31, 33, 35^ Alignment of the Chinese FAdV-4 isolate genome sequences with the available complete genomes of FAdV-4 strains in GenBank showed various nucleotide sequence deletions. The effects of these deletions and sequence differences in various regions of the Chinese isolates' genomes on viral replication and pathogenicity remain to be investigated. However, the fact that strain MX-SHP95 with its truncated ORF19 is highly pathogenic implies that the deletion of ORF19 and/or ORF27 might influence the virulence of the novel Chinese FAdV-4 isolates.

The FAdV capsid consists of three main exposed structural proteins, the hexon, the fiber and the penton base. Hexon is the major capsid protein and contains group-, type- and subtype-specific antigenic determinants against which antibodies are produced.^41^ The molecular classification of FAdVs published to date was based on the hexon gene loop 1 region and the fiber gene.^2, 42^ Consistently, Chinese isolates and isolates K31, 922-1, 09-8846 and 09-584 with the same amino-acid substitution at several positions in the hexon clustered into one group, whereas the previously described non-pathogenic ON1 and KR5 strains and the pathogenic MX-SHP95 strain, which shared common amino acids at these positions, clustered together into another group. This result confirms that the hexon gene can be used to investigate the genetic diversity and molecular evolution of FAdV-4 strains.

The fiber protein is related to virus neutralization, cellular receptor binding, tissue tropism and variations in virulence.^43^ Recombinant FAdV-4 fiber 2 protein has been proven to be a protective immunogen against HHS.^44^ The fiber gene can also be used to differentiate the HHS-inducing FAdV-4 isolates from other FAdV-4 strains.^25^ Here aligning the fiber 1 and fiber 2 amino-acid sequences from the five Chinese FAdV-4 isolates with those of other pathogenic and non-pathogenic strains from different geographical regions enabled us to identify multiple amino-acid substitutions, especially in the fiber 2 gene. Amino-acid substitutions at positions 219 (G to D), 307 (P to A), 319 (V to I) and 380 (A to T) within the fiber 2 protein were conserved in all of the reported FAdV-4 isolates from chickens with HHS in comparison with the non-pathogenic isolates. Two additional putative genetic markers (307P to A, 319V to I) were identified when our sequences were compared with those from a previous study.^33^ On the basis of this information and the fact that HHS-inducing FAdV-4 strains from different geographical regions grouped together, but non-pathogenic strains showed a closer evolutionary relationship in a fiber 2-based phylogenetic analysis, it can be speculated that fiber 2 serves as one primary virulence factor gene. To identify the critical sites responsible for the high pathogenicity of HHS-associated FAdV-4 isolates, a reverse genetic system and further molecular analyses of each gene is required.

In summary, our data demonstrated that FAdV-4 with a novel genotype was the predominant serotype involved in the outbreaks of HHS in China. The findings of the current study have highlighted some of the genetic differences between pathogenic and non-pathogenic FAdV-4 isolates. These data will promote a better understanding of the molecular epidemiology and genetic diversity of FAdV-4 isolates that remain circulating in China and will provide more insights into the critical factors determining the pathogenicity of FAdV-4 strains. Finally, the emergence of the highly pathogenic FAdV-4 with some genomic deviations emphasizes that preventive measures against novel FAdV-4 infections on poultry farms should be implemented in China.

## Figures and Tables

**Table 1:** Predicted ORFs that encode potential proteins found in the genomes of the Chinese FAdV-4 isolates

**Gene**	**Strand**	**Location**	**Number of bp**	**Number of aa**
ORF0	R	478–717	240	70
ORF1	R	790–1314	525	174
ORF1B	R	1485–1808	324	107
ORF2	R	1860–2678	819	272
ORF14C	L	2680–3216	537	178
ORF14B	L	3328–3915	588	195
ORF14A	L	4009–4695	687	228
ORF14	L	4679–5308	630	209
ORF13	L	5342–6142	801	266
ORF12	L	6219–7088	870	289
IVa2	L	7102–8286	1185	394
DNApol	L	8270–12 025	3756	1251
pTP	L	12 030–13 838	1809	602
52 K	R	13 968–15 170	1203	400
pIIIa	R	15 157–16 929	1773	590
Penton base	R	17 001–18 578	1578	525
pVII	R	18 587–18 820	234	77
pX	R	18 992–19 531	540	179
pVI	R	19 635–20 318	684	227
Hexon	R	20 364–23 177	2814	937
Protease	R	23 195–23 824	630	209
DBP	L	23 949–25 430	1482	493
Hypothetical 11.7 kDa	L	25 469–25 777	309	102
100 K	R	25 809–28 970	3162	1053
22 K	R	28 591–29 175	585	194
33 K	R	28 591–28 966; 29 154–29 464	687	229
pVIII	R	29 489–30 232	744	247
U-exon	L	30 177–30 464	288	95
Fiber 1	R	30 463–31 758	1296	431
Fiber 2	R	31 742–33 181	1440	479
ORF22	L	33 234–33 824	591	196
ORF20A	L	33 827–34 093	267	88
ORF20	L	34 244–35 131	888	295
ORF42	R	34 970–35 383	414	137
ORF43	R	35 792–36 460	669	222
ORF28	R	36 816–37 109	294	97
ORF29	R	37 184–37 375	192	63
GAM-1	R	37 357–38 172	816	271
ORF17	L	38 788–39 264	477	158
ORF16	L	39 257–39 667	411	136
ORF19A	R	40 245–42 737	2493	830
ORF4	R	42 831–43 328	498	165

Abbreviations: Amino acids, aa; base pairs, bp; left, L; right, R; open reading frame, ORF.

**Table 2:** Amino-acid differences in the major structural genes from HHS-inducing and non-HHS-inducing FAdV-4 strains

**Genes**	**Strains**^a^	**Amino acids at position**																									
*Penton*		42	45	193	356	370	426	486																			
	HNJZ	P	D	I	V	P	V	T																			
	HB1510	·	·	·	·	·	·	·																			
	JSJ13	·	·	·	·	·	·	·																			
	MX-SHP95	·	G	V	A	Q	I	S																			
	KR5	S	G	V	A	Q	I	S																			
	ON1	S	G	V	A	Q	I	S																			
*Hexon*		164	188	193	195	238	240	243	263	264	410	574	797	842													
	HNJZ	S	R	R	Q	D	T	N	I	V	A	I	P	A													
	HB1510	·	·	·	·	·	·	·	·	·	·	·	·	·													
	JSJ13	·	·	·	·	·	·	·	·	·	·	·	·	·													
	MX-SHP95	T	·	Q	E	N	A	E	M	I	T	V	A	G													
	KR5	T	I	Q	E	N	A	E	M	I	T	I	P	G													
	ON1	T	I	Q	E	N	A	E	M	I	T	V	A	G													
*Fiber 1*		14	28	44	69	70	120	127	154	187	252	263	264	311	330	332	375	384	402	430	432						
	HNJZ	A	S	R	G	S	N	A	R	D	L	H	D	H	H	R	S	I	N	-	G						
	HB1510	·	·	·	·	·	·	·	·	·	·	·	·	·	·	·	·	·	·	-	·						
	JSJ13	·	·	·	·	·	·	·	·	·	·	·	·	·	·	·	·	·	·	-	·						
	MX-SHP95	V	I	P	S	G	D	V	H	N	I	Q	E	R	Q	K	P	L	Y	N	·						
	KR5	A	I	P	S	G	N	A	H	D	I	Q	E	R	Q	K	P	L	Y	H	S						
	ON1	V	I	P	S	G	D	V	H	N	I	Q	E	R	Q	K	P	L	Y	H	S						
*Fiber 2*		11–15	29	219	232	261	300	305–307	319	324	329	334	338	343	344	378	380	391	400	403	406	413	435	439	453	459	478
	HNJZ	ENGKP	A	D	Q	T	T	ANA	I	V	L	A	N	L	NI	T	T	T	G	E	I	S	S	E	A	N	L
	HB1510	· · · · ·	·	·	·	·	·	· · ·	·	·	·	·	·	·	·	·	·	·	·	·	·	·	·	·	·	·	·
	JSJ13	· · · · ·	·	·	·	·	·	· · ·	·	·	·	·	·	·	·	·	·	·	·	·	·	·	·	·	·	·	·
	MX-SHP95	- - - - -	P	·	·	N	·	· H ·	·	F	·	T	T	N	S	·	·	S	A	Q	S	T	·	D	·	A	V
	KR5	ENGKP	A	G	E	S	I	SHP	V	F	V	T	T	N	S	A	A	S	A	Q	S	T	T	D	S	A	V
	ON1	- - - - -	P	G	E	S	I	SHP	V	F	V	T	T	N	S	A	A	S	A	Q	S	T	T	D	S	A	V

a All five Chinese isolates (HNJZ, SDDZ, SXCZ, AHBZ and JSXZ) have the same amino-acid mutations at the locations indicated.
