# Supplementary material for: Genetic characterization of novel fowl aviadenovirus 4 isolates from outbreaks of hepatitis-hydropericardium syndrome in broiler chickens in China
Source: Emerg Microbes Infect. 2016 Nov 23;5(11):e117–. doi: 10.1038/emi.2016.115 (PMC5148019; doi:10.1038/emi.2016.115)
Supplement: Supplementary Table S1 [file emi2016115x1.pdf]

Supplementary Table S1 Primers used to sequence the complete genome of FAdV-4.

Primer name	Nucleotide sequence, 5'-3'	Primer location (bp)[a]
1-F	CATCATCTTATATAACCGCGTC	1~22
1-R	TTACAATCAGTTACAATCGGTTCC	1791~1814
2-F	GCTAGATGAGAACCGTAACGCTGCT	1709~1733
2-R	GGACGAAACACTTCTATTCATCTTG	3496~3520
3-F	CGGGGTAGGGTTCATTGTTGGGATT	3459~3483
3-R	ACCGCCACGACTACACCATCCTCTG	5232~5256
4-F	ACAGAGGATGGTGTAGTCGTG	5231~5251
4-R	TGCCAACAACGCCGCCGAAC	6968~6987
5-F	TAGGGTTTGATAAAGACCGTAA	6915~6936
5-R	AACCCTCAATGCGGCTATGTGG	8701~8722
6-F	CGTAGGAGGGTATCGTAAAT	8637~8656
6-R	CGTGCGCGTCACCCAAAAGCTG	10409~10430
7-F	CTTGCTTAAAGTATCGGTCGTAG	10366~10388
7-R	TCGTAGCCATCTCCACCAATCAGCG	12109~12133
8-F	GGATGGTATCGCTGTTGGAAGTCGC	12037~12061
8-R	GCGTCTTTCTCGTCAGCCGAATCTA	13768~13792
9-F	ATGAGTCTGTAGTCGTAG	13753~13770
9-R	GAAGAAACGAGTCAACATGGAAG	15536~15558
10-F	ACGGTCAAAATAAAGTGTT	15479~15497
10-R	TATCGCTAGCCTTGTTGTCT	17240~17259
11-F	CGGAACAGCATTAAGTATCGC	17178~17198
11-R	GGCGGCGCTGGTCCGTCCTGT	18928~18943
12-F	ATAGCCTTCGTGACTTTGGTA	18877~18897
12-R	TCGGTCTAGGATTCCCTTGATG	20660~20681
13-F	AATGTGGGCGACGGTTGGGT	20613~20632
13-R	TGTTACGGAAGGTGTGCGAGAG	22374~22395
14-F	CTCGGGCAGCATCGCCTACTCAG	22337~22359
14-R	AGAGCTGCTCGTGGCGCATTTC	24093~24114
15-F	CGGTGGTGATCAGCTCGTTC	24040~24059
15-R	TAACCCGACGGATTCGACAT	25778~25797
16-F	GCTCGCTTCGGCTTGAATCTATCTC	25710~25734
16-R	ATCCCGTGGAAGGTGGCATAGTTG	27425~27448
17-F	AAGTGCTCCATCACACCTTCCATCA	27355~27379
17-R	AAAGCAGAGTCGTCGGTCATAG	29093~29114
18-F	CGCTGCGGGATACGGTGTTC	28982~29001
18-R	GACCGCTCGATCGCTGACGTAAAT	30747~30770
19-F	CCTACTGACCCTCAACACCAAAACG	30719~30743
19-R	AAAAGTAGCGATGGGTGTGGAGACG	32535~32559
20-F	CGCTTACGGTCTCCGCCAATG	32441~32461
20-R	ATAGGAGTGCCGACGGAGATA	34184~34204
21-F	GCAGGTAAAGTAGAGAC	34132~34148
21-R	CAGTGACGGGATTAGTA	35365~35381
22-F	ACAGCCTATCGGAATGCG	35302~35319
22-R	CCTACCGGTTTAGTGGAGAG	39022~39039
23-F	AGACCAGGTAGGCAGGTTCAGAC	38965~38987
23-R	GCTGATGATGTAATTGTTGACTGAG	41679~40703
24-F	TAACCAACTGAATCACATGAC	40614~40634
24-R	TCTTTTCCCTTCCGCCTCTC	42359~42378
25-F	TGGCCCTCTCCACGACAAT	42302~42320
25-R	TGAATGGTGTGCGAGGC	43806~43822
26-F	CCTGAAGGAACAATCCAACTTTACC	43670-43694
26-R	CATCATCTTATATAACCGCGTC	45646-45667

[a]Numbers correspond to the nucleotide positions within the ON1 strain of FAdV-4.
