# Supplementary material for: Genetic characterization of novel fowl aviadenovirus 4 isolates from outbreaks of hepatitis-hydropericardium syndrome in broiler chickens in China
Source: Emerg Microbes Infect. 2016 Nov 23;5(11):e117–. doi: 10.1038/emi.2016.115 (PMC5148019; doi:10.1038/emi.2016.115)
Supplement: Supplementary Table S2 [file emi2016115x2.pdf]

**Supplementary Table S2 Isolates and reference strains used in this study**

| Sequence | Strains | Country | HHS[a] | GenBank Accession NO |
|---|---|---|---|---|
| **Genome** | HNJZ | Henan(China) | + | KU558760 |
| | SDDZ | Shandong(China) | + | KU558761 |
| | SXCZ | Shanxi(China) | + | KU558762 |
| | AHBZ | Anhui(China) | + | KU569295 |
| | JSXZ | Jiangsu(China) | + | KU569296 |
| | HB1510 | China | + | KU587519 |
| | JSJ13 | China | + | KM096544 |
| | MX-SHP95 | Mexico | + | KP295475 |
| | ON1 | Canada | - | GU188428 |
| | KR5 | Japan | - | HE608152 |
| | CELO | Germany | - | U46933 |
| | 340 | Northern Ireland | - | KC493646 |
| | A-2A | USA | - | AF083975 |
| | HG | Canada | - | GU734104 |
| **Penton** | HNJZ | Henan(China) | + | KU558760 |
| | SDDZ | Shandong(China) | + | KU558761 |
| | SXCZ | Shanxi(China) | + | KU558762 |
| | AHBZ | Anhui(China) | + | KU569295 |
| | JSXZ | Jiangsu(China) | + | KU569296 |
| | HB1510 | China | + | KU587519 |
| | JSJ13 | China | + | KM096544 |
| | MX-SHP95 | Mexico | + | KP295475 |
| | ON1 | Canada | - | GU188428 |
| | KR5 | Japan | - | HE608152 |
| | CELO | Germany | - | U46933 |
| | 340 | Northern Ireland | - | KC493646 |
| | A-2A | USA | - | AF083975 |
| | HG | Canada | - | GU734104 |
| **Hexon** | AG234 | Mexio | + | FN869969 |
| | K99-97 | Kuwait | + | FN869970 |
| | K1013 | Ecuador | + | FN869972 |
| | Peru53 | Peru | + | FN869973 |
| | K31 | Pajistan | + | FN869976 |
| | C2B | Austria | - | AF339923 |
| | Da60 | Germany | - | FN869971 |
| | 09-8846 | Austria | - | FN869974 |
| | 09-584 | Austria | - | FN869975 |
| | 922-1 | Germany | - | FN869978 |
| **Fiber 1** | K31 | Pakistan | + | FR872891 |
| | AG234 | Mexico | + | FR872892 |
| | K1013 | Ecuador | + | FR872898 |
| | C344 | Chile | + | FR872900 |
| | IV37 | India | + | FR872901 |
| | K88-95 | Pakistan | + | FR872902 |
| | Peru53 | Peru | + | FR872903 |
| | Peru54 | Peru | + | FR872904 |

| | | | | |
|---|---|---|---|---|
| | K99-97 | Kuwait | + | FR872905 |
| | Da60 | Germany | - | FR872895 |
| | C2B | Austria | - | FR872896 |
| | 922/1 | Germany | - | FR872897 |
| | 09-584 | Austria | - | FR872907 |
| | 09-8846 | Austria | - | FR872908 |
| **Fiber2** | K31 | Pakistan | + | FR872909 |
| | AG234 | Mexico | + | FR872910 |
| | K1013 | Ecuador | + | FR872916 |
| | C344 | Chile | + | FR872918 |
| | IV37 | India | + | FR872919 |
| | Peru54 | Peru | + | FR872921 |
| | Peru53 | Peru | + | FR872922 |
| | K99-97 | Kuwait | + | FR872923 |
| | K388-95 | Mexico | + | FR872927 |
| | Da60 | Germany | - | FR872913 |
| | C2B | Austria | - | FR872914 |
| | 922/1 | Germany | - | FR872915 |
| | 09-584 | Austria | - | FR872925 |
| | 09-8846 | Austria | - | FR872926 |

[a]hepatitis-hydropericardium syndrome, HHS
